# The evolution of personality disorders: A review of proposals

**DOI:** 10.3389/fpsyt.2023.1110420

**Published:** 2023-01-30

**Authors:** Fernando Gutiérrez, Francisco Valdesoiro

**Affiliations:** ^1^Hospital Clínic de Barcelona, Institute of Neuroscience, Barcelona, Spain; ^2^Institut d’Investigacions Biomèdiques August Pi i Sunyer (IDIBAPS), Barcelona, Spain

**Keywords:** personality, personality disorders, evolutionary psychology, evolutionary psychiatry, natural selection

## Abstract

Personality disorders (PDs) are currently considered dysfunctions. However, personality differences are older than humanity and are ubiquitous in nature, from insects to higher primates. This suggests that a number of evolutionary mechanisms—other than dysfunctions—may be able to maintain stable behavioral variation in the gene pool. First of all, apparently maladaptive traits may actually improve fitness by enabling better survival or successful mating or reproduction, as exemplified by neuroticism, psychopathy, and narcissism. Furthermore, some PDs may harm important biological goals while facilitating others, or may be globally beneficial or detrimental depending on environmental circumstances or body condition. Alternatively, certain traits may form part of life history strategies: Coordinated suites of morphological, physiological and behavioral characters that optimize fitness through alternative routes and respond to selection as a whole. Still others may be vestigial adaptations that are no longer beneficial in present times. Finally, variation may be adaptative in and by itself, as it reduces competition for finite resources. These and other evolutionary mechanisms are reviewed and illustrated through human and non-human examples. Evolutionary theory is the best-substantiated explanatory framework across the life sciences, and may shed light on the question of why harmful personalities exist at all.

## 1. Introduction

Personality disorders (PDs) have increasingly been considered to be pathologies (1), that is, psychobiological dysfunctions caused by genetic defects, poor parenting, trauma, or a combination thereof (2). This is not an unreasonable claim: All body systems may malfunction, and the motivational, emotional, and cognitive systems that constitute personality are unlikely to be an exception. Moreover, extreme personality traits may impose costs on their carriers or on the people around them, causing affliction and harming every aspect of life, including employment, family, social life, status, health, or personal autonomy (3, 4). In fact, they may place a burden as great as that of many severe mental or physical disorders (5).

This view, however, is not unanimous. The pathological nature of PDs was dismissed at the very outset (6) and remains controversial today: The expected dysfunctions underlying PDs have proven elusive (2), their boundaries with normality are fuzzy (1, 7), diagnosis is heavily influenced by social judgment (8, 9), and the evidence of their harmfulness is mixed at best (10–14).

Also from an evolutionary perspective, the fact that natural selection has been unable to eliminate PDs has been regarded as a paradox (15, 16). The heritability of PDs is reported to be as high as 45% (2, 17). In consequence, one might expect them to be eroded by natural selection at a rate proportional to their heritability and harmfulness (15, 18). The fact is, however, that they remain in the population with prevalences ranging from 9–12% (10, 19), which raises questions about their dysfunctionality.

Evolutionary theory is proving critical for understanding human health and disease, including infections, cancer, and auto-immune diseases (20–22), but attempts to unravel personality and its disorders from this perspective have only just begun (23–25). We now know that personality differences are ubiquitous in nature, from insects to primates, and that these differences are relevant for Darwinian fitness (26–29). For this reason, understanding the evolutionary bases of heritable personality variation has become a major aspiration in evolutionary biology (30). Although apparently maladaptive traits are not uncommon in non-humans, they are routinely conceived as strategies, not disorders (27, 31–33). Therefore, it is not implausible that personality variation is maintained in humans by the same mechanisms as in other species.

This review offers a brief recap of the main principles of evolution by natural selection (section “2. The spread of the fittest”), outlines the evolved action systems that underlie personality in humans and other animals (section “3. Action systems”), and provides a general overview of the diverse mechanisms that can maintain personality variation (sections 4–8). It ends with some remarks on how evolutionary theory can aid the understanding of normal and disordered personalities (section “9. Discussion: What is a personality disorder? “).

## 2. The spread of the fittest

The basic mechanism of natural selection is simple (18, 34). Members of a species differ phenotypically from each other. These differences are partly due to genetic mutations that are continuously emerging anew; they accumulate in each generation, and are transmitted to the offspring. As mutations occur randomly (i.e., they are copy errors), most of them produce harmful or at best irrelevant traits (35). Thus, all variation arises first by mutation, and it is on this variation that natural selection acts. Carriers of disadvantageous traits, say weakened immunity or a slower running speed, will on average die before than their conspecifics, or will produce fewer descendants, with the result that these traits will tend to die out. In fact, small disadvantages can eliminate a character within a few generations (15). In contrast, a minute proportion of mutations produce traits that, just by chance, provide the individual with some advantage over its fellows: For example, a greater ability to metabolize oxygen, a skin that facilitates camouflage, or a greater proneness to look after offspring. The frequency of this trait in the population will increase through the successive generations, and it may eventually replace the wild type. Thus, natural selection is the differential reproductive success of individuals due to differences in certain heritable traits. This success is what we call fitness. Any trait—strength, ability, attractiveness, longevity, health, intelligence, sociability, memory—maintained because of its positive effects on fitness may be an adaptation.

**FIGURE 1:**
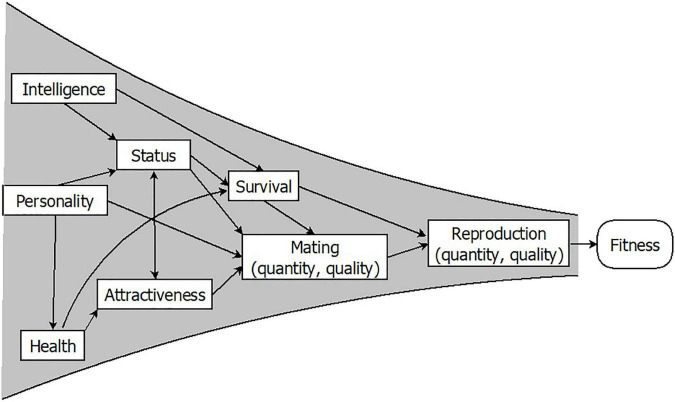
Individual traits must pass through the reproductive success funnel to be evolutionarily relevant. Adapted from Gutiérrez (38) with permission from Siglantana Editorial.

Fitness is most often measured through lifetime reproductive success (34, 36, 37). To ascertain whether a trait enhances fitness, we can assess whether individuals carrying it produce more children over the course of their lives than those who do not. Furthermore, given that other components such as survival and mating success are key preconditions for successful reproduction, they are commonly used as indicators of fitness. If a trait is associated with more or better mates, or with a longer life, we may consider this trait to be adaptive. Finally, organisms differ in a range of traits such as health, strength, attractiveness, intelligence, or certain personality features, which may determine fitness outcomes. However, only when these traits modify the number or quality of the progeny are they evolutionarily relevant. Conversely, any heritable trait leading to differential reproduction will increase or decrease its frequency in the population: That is, it will evolve by natural selection. In essence, selection may be thought of as a funnel, with countless traits having a more direct or remote impact on fitness components, and sometimes having intricate relationships with each other (Figure 1). Only traits whose effect is exerted at the very end of the funnel will have an adaptive significance.

## 3. Action systems

**FIGURE 2:**
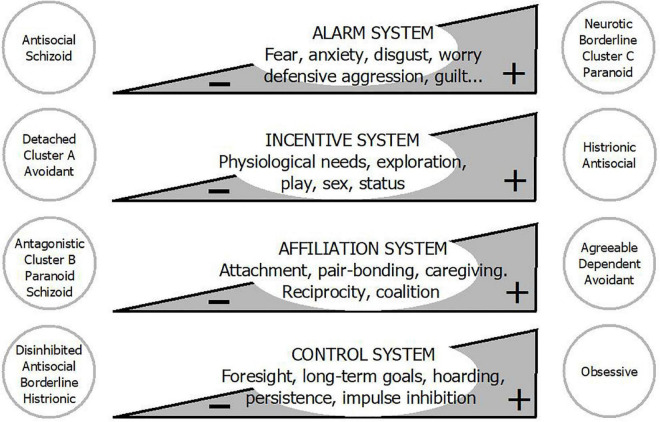
Personality disorders are not qualitatively different from normal personality: They are located at the extremes of basic action systems. Adapted from Gutiérrez (38) with permission from Siglantana Editorial.

Action systems are evolved psychobiological programs that guide organisms’ behavior toward relevant resources and away from menaces (Figure 2). These programs are innate, but are calibrated during ontogeny by tapping into environmental cues (38). Although each one has different triggers and biological goals, and operates independently, they can also activate or inhibit one another. Their ultimate function is to adapt the individual to the environment, maximizing gene transmission. Action systems are probably not mechanisms in a literal sense, but rather overarching categories encompassing narrower-range functionally related systems on whose exact architecture and organization agreement remains incomplete (39–44).

The relative sensitivity and strength of action systems vary among individuals, giving rise to personality differences (45, 46). In fact, action systems can be understood as the dynamic processes behind personality structures (47), with which they show approximate parallelism (40, 46, 48, 49). They also have a conceptual overlap with the main axes of pathological personality, which can be assumed to reflect their hypoactivity or hyperactivity (39, 50–52). Categorial PD diagnoses, which are heterogenous constructs based on clinical observation, may be located at the extreme of one or several systems (Figure 2) (53).

The *alarm system* is designed to react to threats to biological goals *via* automatic defensive responses (40, 46). These consist of diverse aversive emotional states—anxiety, fear, sadness, anger, disgust, guilt, shame, jealousy—attuned to specific mishaps, and behavioral responses such as vigilance, avoidance, flight, freezing, appeasement, or aggression, among others (54, 55). Managing threats is not only necessary for survival; it is probably the main reason why we have a nervous system at all. Despite being a universal device, individuals differ greatly with regard to its sensitivity and strength. While some perceive threats everywhere and live chronically frightened by real or imaginary hazards, others seem unaware of possible damage or loss, and take unwise risks. Negative emotionality (or neuroticism) reflects this variation, with its upper pole covering a range of distress-related traits such as affective instability, anxiety, worry, insecure attachment, mistrust, rage, or self-harm (56). Overreactive defense mechanisms underlie many PDs, especially borderline, avoidant, and paranoid, though the threatening situations differ in each one (abandonment, negative judgment, and betrayal, respectively), whereas schizoid and antisocial personalities show hypoactive alarm systems (53, 57, 58).

The *incentive system* detects resource opportunities calibrated by an individual’s needs, and energizes behavior toward appetitive stimuli (40, 50). Besides homeostatic needs such as food or liquids, it encompasses subsystems aimed at exploring the environment, hoarding material assets, playing, maintaining social contact, having sex, or attaining status (46). Its variation is related to extraversion and positive emotionality (39), but also to impulsive sensation seeking, unrestrained behavior, risk-taking, and disorderliness, which characterize the disinhibition domain and some cluster B disorders (53, 56). Subjects with robust incentive systems experience urgent and absolute necessities and are attracted by any bait disregarding calls for caution, only to forget it immediately and to head for the next one. The hypoactivation of this system, in contrast, defines people who naturally experience few needs and weak motivations, such as detached or schizoid personalities.

The third system, the *affiliation system*, drives us to exchange company, protection, and affection with our conspecifics and to establish enduring bonds, or alternatively makes us indifferent to them. It actually involves a variety of relatively differentiated action systems such as attraction, pair-bonding, care-eliciting, care-giving, or reciprocity (41, 44, 59). These systems, particularly in avian and mammal species, fulfill fitness-related functions such as obtaining protection from attachment figures during growing years, making friends or allies, attracting and retaining mates, or keeping offspring safe. Histrionic, dependent, and borderline PDs may reflect the hyperfunction of some of these affiliation subsystems (53, 58). In contrast, low affiliation is a tendency toward emotional restraint, unconcern for social involvement, and discomfort with intimacy, which is typical of detachment (53). This pole also includes dissocial and antagonistic features, such as low empathy, selfishness, opportunism, distrust, and hostility, which are present in paranoid, narcissistic, and antisocial PDs (57).

Finally, the *behavioral control system* inhibits impulses arising from all the above systems in accordance with the individual’s future interests, such as valued long-term goals or social reputation. If it is weak, it leaves the individual at the mercy of these urges (39, 40, 50, 60). In fine, it makes decisional balances between current and future opportunities and perils (61). Conscientiousness, self-regulation, and effortful control are valued qualities but, when extreme, may lead to the perfectionistic and hardline attitudes that characterize anankastia (62). Per contra, the underactivity of this system implies discounting the future and is typical of cluster B disorders (53, 58).

A further system concerns the dominance-submission axis (63), which is paramount in social species but occupies only a minor place in human personality taxonomies (64). Dominance is characterized by a sense of superiority and self-worth, striving for power, and signaling authority and competence; it is the main feature of narcissistic personalities (64, 65), and is often assigned to the antagonism-dissociality axis. Subordination entails low self-esteem, the need for approval, fear of negative evaluation, and appeasement behaviors; it is related to avoidant and dependent PDs, and is generally subsumed into the negative emotionality domain (66).

As might be expected, action systems are not specific to humans. Other animals not only have personality, but their personality is organized along roughly the same axes as ours (26, 28). Neuroticism and extraversion have been found throughout the phylogenetic tree as far away from humans as fish, octopuses, and insects (67), which means that personality is at least 100 million years older than *Homo sapiens*. Affiliation and dominance systems have been found only in gregarious species, mainly mammals, and control only in higher primates and humans.

## 4. How a harmful trait can still be advantageous

The first reason for the permanence of PDs in the population is that unpleasantness or social undesirability imply neither dysfunction nor low fitness. That is, while clinical adaptation refers to attaining wellbeing and fulfilling socially assigned roles, Darwinian adaptation is just about spreading genes (7, 68, 69). Not only is suffering often irrelevant to fitness, but certain clinical conditions may enhance fitness after all. For example, fertility falls below 50% in affective, neurotic, and psychotic disorders (15), whilst PDs do not cause significant reproductive disadvantages overall (12). On the other hand, PD diagnoses include heterogeneous or even opposite personality patterns, so that taking them as a whole will obscure the fact that some of them definitely increase resource acquisition, deter risk-taking and antisocial acts, multiply the number of mates, or increase reproductive output (11, 12, 14, 70, 71). As a consequence, the idea that PDs are alternative strategies rather than disorders is gaining ground (23, 31). Neuroticism, psychopathy, and narcissism have been widely studied and imply the principal action systems, and so they will be taken as illustrative examples here.

### 4.1. Neuroticism and the alarm system

Neuroticism (or negative affectivity) is probably the most detrimental personality trait ever found (72). It causes unending concerns that comprise reduced wellbeing, relationship troubles, career difficulties, and health problems including psychopathology (13, 73, 74). The repeated enactment of a hyperfunctional alarm system wastes energy, interferes with all other action systems, and produces physiological damage in the long run, resulting in premature death across species (74, 75).

Intriguingly, although recurrent fears and miseries may result from the dysregulation of alarm circuits, they may also be part of their normal, survival-enhancing operation (76–78). The fact that red-flag responses are aversive is an essential part of their utility, as unpleasant emotions mobilize defensive behaviors. Even if we assume that it is their excessive frequency, intensity, or duration that turns them into a disorder, “excess” does not mean the same thing from clinical and evolutionary perspectives. This has been formulated probabilistically in the *smoke detector principle* (68). Usually, responses to threatening stimuli are rapid actions, taken under conditions of uncertainty, which imply asymmetrical errors: Namely, triggering a false alarm is a far less costly error than failing to respond to a real menace. Under these conditions, natural selection reduces not the overall rate of mistakes, but the net negative effect of mistakes on fitness, displacing the trigger threshold toward the less harmful error (79). In consequence, well-functioning alarm systems tend to misfire when nothing harmful is happening.

Despite plenty of evidence to the contrary, certain studies indeed suggest that neurotic traits can lower mortality in some circumstances (78, 80). Improvements in survival may occur through either health vigilance or harm avoidance (81). For example, internalizing dispositions in childhood predict a 3–9% reduction in injury rates in adolescence and adulthood (82), and subjects who are anxious at age 13 reduce their probability of accidental death at age 40 by a factor of six (83). Evidence on more specific forms of threat sensitivity is lacking—for example, enhanced detection of potential foes in paranoid, abandonment in dependent, or disapproval in avoidant PDs (76, 79). In sum, although neuroticism is hardly ever welcome, it may not always be a defect but may be the increased (and therefore costly) activity of risk-averting adaptations aimed at increasing survival (68, 77, 78, 84, 85).

### 4.2. Psychopathy and the attachment system

Psychopathy includes traits such as impulsivity, risk-taking, future discounting, fearlessness, callousness, and non-cooperative tactics (86). In fact, it involves all action systems: A hyperactive incentive system, along with weak alarm, affiliation, and control systems (87). However, it is its opportunistic interpersonal strategy that has attracted the most attention. Interestingly enough, whereas the search for the deficits behind selfishness and lack of empathy is ongoing (88), what has truly puzzled evolutionary biologists is the existence of altruism and empathy in living creatures (89). Indeed, exploiting or harming others is often not detrimental for the individual, and can constitute an effective (though risky) way of enhancing one’s own fitness (90). Far from being diseased, some psychopaths seem finely designed to trap prey (91). For example, like many predators, they are able to use the prey’s gait to estimate its vulnerability (92).

However, the strongest card of psychopaths regarding fitness has been deemed to be their promiscuous, uncommitted, and opportunistic mating strategy, purportedly aimed to gain reproductive benefits (93–96). Rather than being a rarity, unrestricted sexuality is almost universal in nature including our own phylogenetic branch, as 93% of mammals are non-monogamous (59). Furthermore, many people find psychopaths alluring, and traits such as novelty seeking, low empathy, or disinhibition boost the number of mates (12, 14, 94). More specifically, though both sexes prefer risk avoiders for long-term relationships, risk takers are favored for the short-term (97). This is not exclusive to psychopaths: Cluster B subjects as a whole also turn out to be more attractive to the opposite sex (71, 98, 99), and triple the number of sexual partners (12, 70, 100). Though cluster B subjects have been shown to out-reproduce their low-B counterparts (12, 101, 102), whether psychopaths ultimately have greater fitness in reproductive terms is less clear. Greater reproductive success may be offset by poor parenting (103, 104). Furthermore, legislative changes and effective birth control appear to have partially uncoupled mating success from reproduction (14, 105). Even so, some evidence suggests that reproduction at the expense of others may still be the successful strategy it was ancestrally (93, 106).

### 4.3. Narcissism and the dominance system

Although narcissism shares with psychopathy its mating strategy (94, 99), it is particularly characterized by its striving for escalating the hierarchy of status, power, or fame (65). Hierarchy formation is ubiquitous among social species. Contrary to appearances, it reduces conflict by resolving problems of allocation of limited resources, within-group discord, and collective action (63, 107, 108). Humans who do not previously know each other rapidly and spontaneously self-organize into a hierarchy, and this is so from the age of three (109). Rank is partly determined by personality traits of dominance and subordination, which are signaled to others through cues such as size, formidability, self-confidence, initiative, voice pitch, facial expression, or body postures, depending on the species (110–112). A fierce struggle for status is not pathological in nature, though it does entail costs, such as the energy devoted to aggressively maintaining rank or a shorter lifespan in some species (113, 114). In humans, narcissism and dominance also tend to bring social discord, but above all they cause distress to others (115, 116).

Narcissists not only crave high status but, unexpectedly for a disorder, quite often achieve it (11, 117), in the form of charismatic leadership (118, 119), job level (11, 112), income (120, 121), and popularity (122). Status, once achieved, provides huge benefits for the holder (123–126), and many of the advantages associated with narcissism may come in this way (117). For example, unlike psychopathy, narcissism is a buffer against health problems and premature death (127). Longevity may increase not only owing to material welfare, but also to the psychological consequences of high status (128). Notably, Nobel Prize winners live longer than just nominees, and graduates longer than poorly educated people (129, 130). Status multiplies the number of mates in men, and these mates are younger and more attractive (131–133). It has historically enhanced fertility as well (134–136), though this is less clear since the demographic transition (137) or in women (133, 138).

Interestingly, accession to high rank may also trigger a feed-forward loop of dominant and narcissistic traits (139). There are increases in self-esteem, assertiveness, tolerance of stress, executive functioning, creativity, and disregard for others (125, 140). Serum levels of serotonin and testosterone increase within days or weeks and profound changes in neural activity are triggered (141–143). These changes make retreat during fights less likely, and increase the chances of further escalating the hierarchy (144). But even the most bothersome features of narcissists, such as the will to hang on to power or to regularly receive recognition, may be part of the normal functioning of the power pyramid across species. For example, some male crayfish (*Procambarus clarkii*) are sore losers that will rather die than giving up their hierarchical position (141), and dominant treeshrews (*Tupaia belangeri*) stop eating and fighting back after defeat, and die from renal shutdown within 2 weeks (145). In an iconic experiment about claiming recognition, the serotonin levels and humor of alpha-male vervet monkeys (*Chlorocebus pygerythrus*) collapsed when they stopped receiving submissive signals from subordinates (146), though they recovered on fluoxetine as also occurs in humans (147). Narcissism may then be a high-risk high-reward strategy that pushes individuals to the apex of the status hierarchy if it succeeds, but crushes them if it fails (64, 148). In the end, an adaptive trait does not need to always succeed—only on average.

## 5. Variation maintained despite natural selection

**FIGURE 3:**
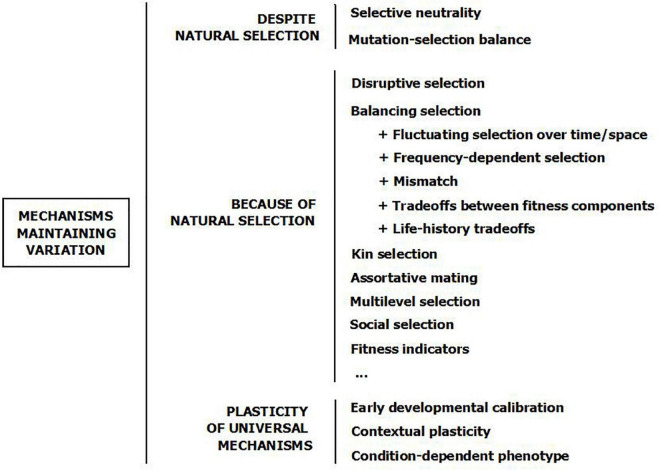
Evolutionary mechanisms which are able to maintain variation in behavioral traits. The mechanisms are not mutually exclusive. The schema is based on (29, 44, 69, 156, 159, 160).

Showing that a clinically maladaptive trait may actually be beneficial for fitness is not the same as explaining variation. In accordance with the above, we could expect these advantageous traits—anxiety, promiscuity, or ambition—to give the highest payoffs and then spread in the population, displacing less successful alternatives (149–151). On the contrary, the norm in nature is variation (152, 153). Why and how individual differences are maintained is unknown, but a number of evolutionary mechanisms have been held to be able to maintain trait variability in the population (Figure 3) (29, 30, 149, 152, 154–160). Some of them assume that variation is maintained not because of natural selection, but in spite of it. Human and animal examples may be used indistinctly by way of illustration, as these mechanisms are thought not to differ between species.

### 5.1. Neutrality

Individual differences in personality were initially regarded as mutational noise around an adaptative peak of optimal functioning (161). This variation was considered to be inconsequential for fitness and therefore invisible to selection, meaning that it cannot be removed. The weakness of this proposal is that personality *is* consequential (73, 74). In fact, personality has been shown across species to bear upon central components of fitness such as survival, mating, and reproduction (16, 27–29, 73, 74, 154, 156, 162–164). For this reason, selective neutrality is no longer considered a plausible explanation for personality variation (16, 165).

### 5.2. Mutation-selection balance

Nevertheless, variation could be maintained by random mutations which are mildly detrimental, with the result that natural selection is unable to remove them completely. Each human being inherits around 70 new germline mutations, though with large differences between individuals (166). These mutations are far more likely to be deleterious or neutral than beneficial (15, 35, 157). As mental traits are determined by thousands of genes (indeed, half of human genes code for the nervous system) many of these mutations will affect brain functioning, and so the mutational target size is immense. On the other hand, each gene accounts for only a very small variance (167). Both facts combined cause natural selection to be incapable of purging mutations, with the result that they may persist for generations (15). Even traits under strong purifying selection can maintain abundant genetic variation if the target is large enough. The total burden of the remaining deleterious mutations is called *mutational load*, and it varies from one individual to another and determines the probability of maladaptive traits.

Although there is some consensus that the mutation-selection balance has a role in low intelligence and attractiveness, poor health, and major mental conditions like schizophrenia or bipolar disorder, it does not fit personality variation equally well. One source of evidence is fitness itself: Major psychiatric disorders harm all fitness components at once (15, 16), but no net effect on mating or reproductive success has been found for PDs as a whole (12). Also *paternal age*, which predicts the number of new genetic mutations and is used as a proxy for mutational load (168), supposes a risk for schizophrenia, autism, bipolar disorder, and intellectual disability, but not for PDs (157). As for *fluctuating asymmetry*, it is the random deviation from perfect bilateral facial or body symmetry, and is assumed to reflect the inability of an organism to buffer developmental perturbations caused by mutational load or environmental insults (169). Fluctuating asymmetry correlates with intelligence and with infectious and mental disorders (155, 170), but not usually with personality traits (169, 171). When it does correlate, it is extraverted, aggressive, and risk-taking individuals who show the highest symmetry (172). Finally, *inbreeding*—the production of offspring by consanguineous parents—exposes recessive mutations to higher rates of homozygosity (173, 174), so that deleterious traits linked to condition are more likely to be expressed with damage to fitness (*inbreeding depression*) (175, 176). Inbreeding increases the risk for uni/bipolar depression, and has shown small yet significant associations with certain personality traits: Increased harm avoidance and schizotypy, and reduced affiliation and novelty seeking (177, 178). However, well-powered samples have not confirmed its association with neuroticism (179).

## 6. Variation maintained because of natural selection: Balancing selection

In the last 30 years the notion that variation may be maintained by selection has gained ground. However, the most frequent types of selection in nature are *directional selection* (180), which pushes the trait mean toward one of the extremes, and to a lesser extent *stabilizing selection*, which favors intermediate values and selects against the extremes, as is the case with many morphological traits. Neither of them is able to maintain variance in a trait; in fact, both tend to erode it (18, 34). Though a third type, *disruptive selection*, does favor extreme values over average values and may maintain variation, it is surprisingly infrequent in nature (181).

However, directional selection on a trait is not always homogeneous (182). Instead, it may be inconsistent over time, across different environmental conditions, or for different components of fitness. These conflicting pressures may shape complex evolutionary dynamics (called *balancing selection*) that result in divergent responses to environmental challenges, and hence in interindividual variation (183, 184). In fact, balancing selection is common in nature (156), and is the most frequent explanation for the maintenance of behavioral variation (16, 48, 184, 185). The key concept here is that there is no single solution to the problem of perpetuating our genes.

### 6.1. Fluctuating selection over time and space

Traits may turn out to be advantageous at a given time or place, and not at others. Consequently, the strength, direction, or form of selection changes or reverses periodically due to environmental heterogeneity, and no level of the trait outperforms others outright (36, 48, 156, 186). These shifts have been reported to be frequent (182), and may respond to fluctuations in temperature, resource availability, predatory or parasitic pressure, or sex ratio, among many other factors (187). In a classic example, the boldest and most aggressive female great tits (*Parus major*) survive more than fearful ones in harsh years, in which exploring new territories is necessary, but the reverse is true in years of plenty, when high population density increases aggressive encounters between bolder individuals (188). Thus, annual fluctuations in the abundance of resources cause opposing selective pressures that cancel each other out, resulting in no net selection on the trait and the maintenance of a shy-bold axis in the population (189). Also, in the guppy fish (*Poecilia reticulata*), vigilance and escape are lost in low-predation environments, suggesting that maintaining an alarm system imposes heavy costs. However, after experimental reintroduction into a high-predation environment, the down-regulation of these defenses undermines survival, so that escape ability evolves again in about thirty generations (190). Overall, high neuroticism yields larger payoffs in dangerous environments but seems to be disadvantageous otherwise. Depletion of boldness, activity, and exploration under high predatory pressure has been extensively documented across species (191–193).

The same kinds of tradeoff may operate in humans, though data are limited here. For example, personality traits such as industriousness, extraversion, prosociality, and neuroticism produce reproductive benefits in Tsimane women living near towns in Amazonian Bolivia, but costs in those living in the forest (194). Also, although there is no relation of conscientiousness and openness with fertility in cohorts born in 1920, an increasingly negative association has developed throughout the twentieth century (195). Finally, though self-control is advantageous in resource-rich environments, it may not be in dangerous or highly variable environments, despite the long-term costs of impulsivity (196). In addition, environmental variation over time has been found across species to lead to a diversifying “bet-hedging” strategy, which spreads the risks producing a random distribution across trait levels. No matter how the environment changes, a part of the offspring will be well fitted (29, 197).

### 6.2. Frequency-dependent selection

A particular instance of fluctuating selection is negative frequency-dependent selection, in which a trait produces higher fitness payoffs the less frequent it is in the population (198–200). Environmental heterogeneity is, in this case, the momentary prevalence of the trait itself. Negative frequency-dependent selection is common in natural populations, and is thought to be a major contributor to the maintenance of phenotypic variation (201). In coho salmon (*Oncorhynchus kisutch*), as in many fish and insects, large and dominant males fight each other to gain access to fertilizing females’ eggs, whilst small males hide behind rocks and take advantage through sneak fertilization. The populational proportion of “sneakers” self-regulates: When they are few in number, they benefit from cost-free reproduction and increase their numbers, but at higher prevalences they get in each other’s way and lose their advantage, with the result that their numbers fall (202). In essence, statistically rare strategies can take a fitness advantage of exploiting a part of the resource spectrum for which competition is weaker, in a process known as *ecological release*. This mechanism has been proposed as an explanation of the presence of psychopathic individuals at a constant prevalence under 3–4% in many social species, including humans (93), but it may also explain the maintenance of personality variation more generally (203). In essence, a free-rider would be fitted just because all others are cooperators, and a bold individual because all the rest are shy. As a result, different adaptive tactics coexist at evolutionary equilibrium within a population (189, 204). Many interactions, however, may imply three or more tactics in equilibrium, as in the so-called rock-paper-scissor dynamics, whose mathematical basis derives from game theory (198, 201).

### 6.3. Mismatch

Sudden changes in environmental conditions can decrease the fitness returns of a previously well-suited trait, resulting in an *ecological trap* (205). Typically, changes are due to human activity, such as habitat transformation, technological advances, culture, or urban lifestyles, and are so rapid that they exceed a species’ capacity for genetic adaptation. When trapped, organisms take decisions that reduce their survival or reproduction based on cues that formerly increased fitness but are now mismatched with the current environmental conditions (206). This is the case of seabirds that choose to eat floating plastic over fish, or insects that lay their eggs on the asphalt instead of the pond surface. The transition to modernity is also changing the direction and intensity of natural selection acting on human traits. For example, the same yearning for fat and carbohydrates that pushed us to seek game and fruit in the recent past now points us in the direction of fast food and pastries, sparking an obesity epidemic (207). Hyperactivity and wandering attention might be advantageous in hostile natural environments, but became a disorder after the implantation of compulsory schooling in the twentieth century (208). Contraceptives and legislative changes seem to have hampered the uncommitted reproductive strategy of psychopaths by delinking mating success from reproduction (14, 105). Our affiliation systems appear to be poorly prepared for managing social isolation, dissolution of family bonds, and increased social competition (209). For their part, shy people deal with hundred of strangers in large urban areas instead of a small group of relatives (210). Thus, our action systems are perfectly adapted to the past, but are triggered by cues that are now outdated.

### 6.4. Trade-offs between different components of fitness

It follows from Figure 1 that the different components of fitness (survival, mating, reproduction, and parenting) do not necessarily work in unison. Although some traits, say intelligence or physical condition, might favor all of them at once, others turn out to be successful because of their impact on a sole component, even if it harms all others (211, 212). Diverging strategies could yield similar fitness payoffs in the end, thus maintaining diversity within a population (156, 213). If a trait is involved in a trade-off, natural selection cannot deplete its genetic variance.

An iconic example is the peacock’s train, which perplexed evolutionary biologists for decades. If natural selection cleans out maladaptive traits, we may wonder why peacocks haul a tail measuring five feet long that increases visibility and hinders flight, thus augmenting the risk of predation. The existence in nature of colossal horns, garish colors, and deafening songs seems at first glance to represent both a waste of energy and a deadly challenge. As Darwin suggested, these traits are simply aimed at attracting mates, and so are subject to sexual selection. The exhibition of epic ornaments or risky behaviors unequivocally signals to potential partners or competitors the genetic quality and good condition of the individual (214, 215). This is the *handicap principle*: Signals are reliable precisely because of their prohibitive cost, as a less gifted individual cannot develop or maintain such ornaments, just as most people cannot afford a 65-m yacht (216, 217). Strong sexual selection may sometimes compromise survival (214, 218). However, mating success impacts on reproductive output more directly than any other component of fitness and can spread traits even at the cost of increased mortality (180).

Sexual selection may have a stronger role in personality maintenance than previously thought (219). For example, having a bold personality incurs a survival cost in a range of species but, in exchange, it increases mating success, so that a shy-bold axis of variation is maintained in the population (28). This mechanism has been described in humans (220). Whereas extraversion is associated with indicators of premature death such as hospitalizations due to accident or illness, it also leads to higher sex frequency, more mates, and a greater inclination toward short-term mating and extra-pair affairs (221, 222), as well as to more children (162, 164, 221, 223–225). By contrast, conscientiousness enhances survival (74, 226), but may make missed opportunities more likely, e.g., regarding mating (48).

Another strategy in equilibrium possibly is the “crazy bastard” syndrome, applied to young men who impress friends and potential mates, and intimidate rivals, through voluntary physical risk-taking (227, 228). This is designed to signal their good physical condition, bravery, and dominant position among peers, and may include driving at full speed, taking drugs, locking horns for trivial reasons, or breaking the rules in a thousand imaginative ways. The syndrome is universal among human males, emerges at the beginning of reproductive age, and smooths (hopefully) in adulthood. Although the costs are huge in the form of peak juvenile deaths (227), this syndrome is ultimately associated with more mates and a higher group status, so it is considered a sexually selected complex (97, 229). As already mentioned (section “4.2. Psychopathy and the attachment system”), similar tradeoffs can apply to psychopathy and cluster B disorders, in which subjects excel in the mating arena at the price of a disproportionate exposure to physical risks (14, 82) and reduced survival (226). In contrast, cluster C subjects are better-safe-than-sorry strategists who are willing to give up on opportunities in return for avoiding perilous situations (12).

### 6.5. Life history tradeoffs

Life history theory provides a broader picture of the tradeoffs between the components of fitness. It considers that these tradeoffs are not independent of each other but correlate, and approaches them as a whole (185, 230, 231). The underpinning assumption is that the energy available for each organism is limited, so that all fitness components—growth, quantity and quality of mates, quantity and quality of offspring, parenting, body maintenance, longevity—cannot be optimized at once. Rather, each investment detracts from others, so that “choices” are obliged. For example, either promiscuous mating or having large numbers of progeny impact negatively on offspring quality in humans and other large mammals (232). Thus, life histories essentially are about how energy is allocated across the life course between growth, survival, and reproduction, giving rise to a range of strategies that are aimed at optimizing fitness through different pathways and that coexist within the same population.

The best-studied life history strategies are those that shape the fast-slow axis (233, 234). The fast strategy characterizes rats: They are short-lived, grow quickly, have many offspring but invest little in them, and have high pup mortality. All these features lead to rapid population growth. Elephants, on the other hand, are slow strategists: They are long-lived, reach maturity late, have only one calf but invest heavily in it, have low calf mortality, and expand slowly (235). Most species fall somewhere between the fast and slow poles (236). Two recent developments make life history theory relevant to PDs. First, life histories not only differ between species, but also between individuals within a species, our own included (237, 238). Second, personality may play a key role in life history choices, both in humans (50, 239–241) and in other animals (211, 237, 242). For example, humans live long lives or die young, accumulate or spend resources, have many or no mates at all, have many or no offspring, invest heavily in their offspring or vanish after fecundation… Most crucial life history “decisions” are behavioral in nature, and require different underlying motivational, emotional and cognitive machineries, that is, they require different personalities. It follows that personality traits are packaged into broad suites of coordinated morphological, physiological, and behavioral characters (27), and that it is not traits but the entire frame that responds to selection (184, 213, 240, 241).

In humans, conditions such as attention-deficit/hyperactivity disorder, bulimia, impulse-control disorders, and borderline and antisocial PDs have been related to fast life histories (23, 240, 243–246). Strategies at the fast pole of the continuum are believed to maximize fitness under adverse environmental conditions by prioritizing current over future reproduction, mating over parenting, and quantity over quality. Indeed, individuals showing externalizing traits are not well equipped for retaining long-term partners, raising children, or preparing for the future, but they are for short-term mating or opportunistic gains (12, 104, 247). Per contra, anxious temperaments, conscientiousness, agreeableness, autism spectrum disorders, depression, anorexia, and obsessive-compulsive traits have been related to the slow pole (240, 244, 245, 248). That said, simplistic pictures should be avoided. In the field of human personality, externalizing, sociopathic, or sexually unrestricted personality features have too often been regarded as equivalents of fast strategies (203). This does not stem from life history theory, which is based solely on biodemographic indicators (249, 250). In fact, fast features such as early life reproduction and increased reproductive output are also associated with persistence, industriousness, and religiousness (247, 251), so the evidence should be interpreted with caution. Furthermore, it has also been suggested that fitness tradeoffs might be less stable and more complex than previously thought (231, 252).

## 7. Variation due to selection for plasticity: Reaction norms

The fact that a mechanism has evolved does not mean that it is genetically determined (253, 254). Plasticity is ubiquitous in nature, and action systems—and hence personality—are environmentally calibrated over the course of the entire lifespan (164). Thus, it is not only the trait’s value that can be genetically preprogrammed, but also the trait’s capacity to respond plastically to distinct external conditions that modify that value. Interaction with specific features of the environment is in fact critical for the normal development and activation of most evolved adaptations. Each trait actually represents a *reaction norm*: the range of possible phenotypes that a single genotype can produce along an environmental gradient (255–257). Whereas some traits are canalized—the phenotype is kept constant for a given genotype irrespective of the environment—others show broad reaction norms (164, 257). Plasticity extends the range of conditions under which organisms can survive and reproduce, and is thus a buffer against low fitness and extinction (258). However, it is probably not without costs and constraints, so that a balance between plasticity and canalization exists (27, 259). Besides contributing to trait variation, plasticity is itself a heritable trait (260, 261) which differs between individuals (262–264).

Plasticity can take several forms, which partially overlap: Early developmental calibration, contextual plasticity, and condition-dependent phenotype (263). All of them have in common the fact that distinct inputs alter the expression of a universal mechanism, producing individual differences. They differ in the life period in which they operate, in the particular environmental stimuli that trigger phenotypic change, and in their reversibility (149, 263).

### 7.1. Early developmental calibration

Also referred to as developmental plasticity, early developmental calibration denotes the ability of organisms to adjust their phenotype to environmental conditions experienced during ontogeny (265). Developmental events channel individuals into one of several alternative adaptive paths specified by evolved decision rules (253, 266, 267). Changes are made early in life, involve molecular epigenetic processes (268), and are often irreversible (254, 257, 269). The Predictive Adaptive Response model proposes that the early environment provides cues regarding future life conditions, and developmental pathways are modified accordingly (270–272). In mammals, the best route for such a forecast may be *via* the mother (273). For example, vole pups (*Microtus pennsylvanicus*) born in the autumn have thicker coats than those born in the spring, and this depends on maternal hormonal signals during gestation that are contingent upon day length (274). Plasticity also has costs, as it will lead to fitness benefits if the predictive adaptive response correctly anticipates forthcoming conditions, but to mismatch if anticipation fails (259).

Differences in personality and in life-history strategies may be partly due to differences in developmental histories (262, 265, 272, 275). For example, guppies (*Poecilia reticulata*) living in high-predation areas display faster life histories, including quicker growth, earlier age at sexual maturation, and larger litter size (276). Also in humans, the quality of parental care-giving may be a hint of how harsh the future environment will be. External conditions such as family disruption, the absence of the father, the presence of a stepfather, high local mortality, deprivation, unpredictability, and other indicators of environmental threat can calibrate the life-history strategy, accelerating the growing rate and determining adult reproductive tactics (277, 278). Some of these factors are able to advance age at menarche (239, 279), which in turn is a predictor of earlier sexual debut, sexual risk-taking, earlier pregnancy, and larger numbers of children (280–283). Faster strategies have mostly been associated with personality features such as discounting the future, impulsivity, novelty seeking, risk-taking, and social deviance, as well as mistrust, opportunism, egotism, and callousness (38, 239, 277, 278, 284, 285). By contrast, the same fitness-maximizing algorithm calibrates our strategies toward the slow pole when trusting others and preparing for the future can produce a reproductive gain. From this perspective, it has also been suggested that individual differences in neuroticism may result from conditional adaptations, that is, the calibration of the alarm system during development in response to favorable or adverse experiences (85, 240, 286). Hyperreactive defenses are considered to be due not to dysfunctional processes, but to adaptive mechanisms that try to make the best of a bad job (287). In fact, harsh environments and high extrinsic mortality may not be a radical departure from normal rearing conditions (and thus something able to disrupt neurobiological systems) but the usual scenario that human children have historically faced (38, 288). In any case, caution is required in interpreting the evidence at this stage. On the one hand, it is difficult to separate the effects of adverse environments from those of heritable vulnerabilities running in families (289); on the other, these processes are bidirectional, with children being molded by, and at the time actively shaping, their own developmental niche (290).

### 7.2. Contextual plasticity

The ability to facultatively match to the environment does not end in adulthood. When subjects occupy an environmental niche for a while, they tend to behave in stable ways that give the impression of a trait (291). This is also referred to as *stable situational evocation*, and is assumed to be reversible and dynamic (156, 257, 263). For example, cooperation and agreeableness are lower in people living in slums and mountain areas (292, 293), aggressiveness decreases with latitude (294), and having a job or a romantic relationship increases emotional stability and conscientiousness (295). Thus, action systems are programed to attune with the requirements of present socioecological niches throughout adult life too (29, 291, 296, 297), and are responsive to major life transitions and events (298). It has even been postulated that the diversity of personality profiles actually reflects the diversity of existing niches, both in humans and in other animals (299).

That said, socioecological niches are not chosen at random. Owing to genetically driven preferences, organisms try to expose themselves to the selection pressures that suit their traits best, a strategy known as *niche construction* or gene-environment correlation (300, 301). Specifically, individuals *select* (or avoid) certain environments and individuals over others, spontaneously *evoke* certain responses in others, and purposefully *manipulate* their physical and social environments (302, 303). In animals, this includes building nests, choosing richer habitats, or altering physical and chemical conditions. In humans, many apparently uncontrollable experiences and environmental conditions have been proved to be under genetic influence (304). In fact, contextual plasticity is particularly potent in our species, as it involves the social transmission of cultural knowledge, giving rise to phenomena such as ecological inheritance and gene-culture coevolution (305). Thus, genes and environment exert a reciprocal influence through non-linear dynamics whose study requires integrative models (2, 306–308).

Importantly for PDs, niche selection may produce feedback loops that result in exaggerated or apparently maladaptive traits (306). For example, in domestic fowls, crayfish, or humans, dominant traits and status are known to feed each other in an upward spiral that magnifies initial dispositions (139, 144, 309, 310). Highly neurotic people experience more negative life events, which in turn reinforce their neuroticism (311). The proposed mechanism in this case is *adaptive sensitization*: Repeated experiences of distress are taken as a sign that mild alarm responses have been insufficient to protect the organism against threat, and so the trigger threshold is lowered (69). Similarly, individuals at risk for borderline PD are more likely to undergo the life events—break-up, violence, sexual assault—that can set off borderline symptoms (312–314).

Finally, there are also broad differences in the extent to which individuals are influenced by environments and respond plastically to them (i.e., gene–environment interactions) (256, 315–317). There are even individual differences for different types of plasticity (264). Furthermore, often life experiences do not occur in isolation. Events or environmental conditions can by themselves trigger domino effects that propagate and amplify misfortune through feedback loops, embedding it even over generations (128, 318).

### 7.3. Condition-dependent phenotype

A trait may produce costs or benefits depending on other individual features such as strength, intelligence, skills, age, or attractiveness. In this case, the trait may be not selected directly, but is facultatively calibrated to these organismal features taking them as input, in a process known as *reactive heritability* (159, 161, 319, 320). The leading trait is most often quality or condition, the ability to efficiently convert energy into fitness-enhancing traits and outcomes. For example, high-condition individuals are usually bolder across species (318), and high-condition females are choosier regarding potential mates (321). In zebra finches (*Taeniopygia guttata castanotis*), unattractive males place the greatest effort in parenting, whereas attractive males accrue fitness gains through decreased parenting and increased extrapair fertilization (322). Similarly, strength and attractiveness are correlated with extraversion and low neuroticism in humans (319, 323) as well as with men’s (but not women’s) orientation toward uncommitted mating and promiscuity (324). The proposed mechanism is that extraversion and promiscuity render more benefit in attractive than in unattractive individuals, causing positive feedback mechanisms (318). Finally, height, strength, and formidability are related to dominance and aggressiveness in males (325–328), and partly explain sex differences in fearfulness (329). Other evidence suggests, however, that it is aggressiveness that precedes physical strength (330), meaning that physical aggression and formidability may actually have coevolved as part of a sexually selected complex (231). Narcissism, psychopathy, and dark traits overall also have shown small but positive correlations with height, bulk, and attractiveness (99, 331–333), which would suggest that they are facultatively calibrated to condition. Traits will show apparent heritability that must actually be attributed to condition.

## 8. Other selective mechanisms maintaining variation

We will now look briefly at certain other mechanisms that have been proposed. *Kin selection* (89, 334) rests on the fact that organisms are not really able to replicate themselves, but only to produce fairly similar copies. It is genes that replicate, and they can do so for millions of years using living organisms as vehicles (335). Consequently, genetic transmission may also be maximized through *inclusive fitness*, the successful reproduction of relatives with whom we share genes. For example, it has been found that the same genes that lead to schizophrenia produce schizotypal traits in relatives which increase divergent thinking, creativity, and mating success (336–339). This could maintain risk alleles in the population.

*Assortative mating* is the non-random coupling of individuals based on resemblance. It is common in non-human animals (340), but humans also mate assortatively according to age, height, race, education level, and personality traits (341, 342). Regarding personality, the strongest concordance has been found for sensation seeking, psychopathy, Machiavellianism, and narcissism (343–346). This would produce homozygosity for these traits and, consequently, more extreme presentations in the progeny.

*Multilevel selection* reflects the assumption that selection pressures act at different levels of organization—gene, cell, organism, kin, group—depending on the context (347). This mechanism has been invoked to explain the unparalleled levels of altruism in humans (348), but also conditions such as attention-deficit disorder or insecure attachment. Both would bring advantages for the group, such as increased exploration and risk assumption in the former, and greater awareness of threats in the latter (208, 349), even if they are individually impairing.

*Social selection* is based on the fitness gains due to differential success in social competition (253, 350, 351). Due partly to their personality features, individuals can be preferred as friends, allies, partners, employees, or providers, and thus obtain more resources and help (352, 353). In this context, sexual selection may be a particularly relevant type of social selection. It has been hypothesized that humans have acquired their prosocial traits through social domestication (354), in much the same way as wolves became dogs. That is, humans have lost aggressiveness and gained affability through the choices of other humans (350). This theory is not at odds with the existence of selfish and antagonistic individuals, since a cooperative milieu is precisely the environment where free-riders can evolve (93).

*Fitness indicators* theory extends the role of sexual selection in proposing that many human features—intelligence, moral values, creativity, humor—are not indispensable for survival. Instead, they evolved for courtship, just like the peacock’s tail (355, 356). They are complex traits that depend on large parts of the genome (the “*genic capture*” hypothesis) and are thus reliable fitness indicators for potential mates (152, 173, 175). This is the flip side of the mutation-selection balance, since fitness indicators actually signal the absence of mutational load. For example, personality traits such as agreeableness, conscientiousness, or low neuroticism have been said to confer benefits on the carrier and to be universally preferred in prospective mates, so they could be considered to be fitness indicators (357, 358). However, humans are strategic pluralists in the mating arena (217, 359), and these preferences have been found to be reversed in a wide range of circumstances, e.g., when women have psychopathic traits themselves, are looking for short-term relationships, are living in a harsh environment, or are in their fertile period (344, 346, 360, 361). This would rather support a balancing selection scenario.

**FIGURE 4:**
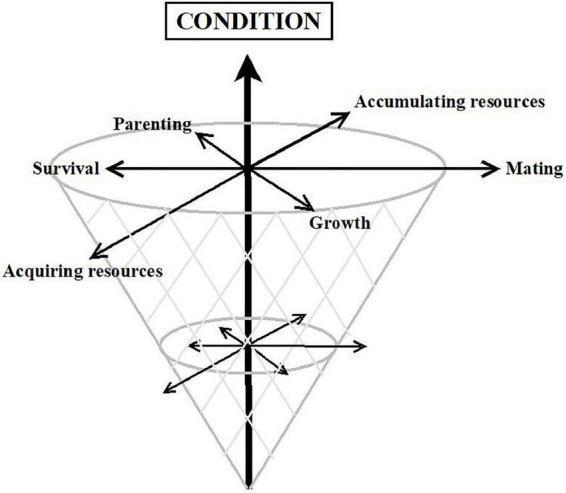
Cone model reflecting condition on the vertical axis and alternative strategies on the horizontal axes.

The array of mechanisms considered here (Figure 3), together with some others such as correlated selection (362), Red Queen processes (363), Fisherian runaway (364), or manipulation by pathogens (365), are not mutually exclusive. Each one may be relevant for distinct traits, or its relevance may vary across sex, time, place, or condition. Furthermore, several of them may act simultaneously or sequentially on the same trait (16, 29, 48, 154, 155, 163). We do not know, however, which evolutionary processes are at work in each case. There is some agreement that traits unidirectionally linked to fitness—such as intellectual disability, unattractiveness, or serious mental disorders—reflect condition, that is, how much energy and resources individuals have available to invest in fitness-related tasks. These traits would fit a mutation-selection balance model better (355) (Figure 4, vertical axis). In contrast, most personality traits rather seem to be related to how the available energy and resources are strategically allocated to different tasks; hence, they fit better with a balancing selection model in which fitness is attained through different routes (16, 149) (Figure 4, horizontal axes).

## 9. Discussion: What is a personality disorder?

We have come to believe that being balanced, outgoing, warmhearted, and industrious is “normal,” while being abusive, cowardly, oversensitive, unsociable, or unhappy are dysfunctions. This is occasionally true and, in fact, some evolutionary approaches see “normal variation” as small maladaptive departures from optimal design (165). However, PDs have suffered a process of pathologization (366), while in fact the evidence thus far rather suggests that many intense personality traits might be fully functional (even if socially reproved) alternative strategies (16, 31). On this basis, evolutionary theory may contribute to redrawing the boundaries between disordered and normal personalities, which remains a contentious issue (1, 7, 8).

Two points need to be stressed. On the one hand, what is normal in nature is variety (28, 29, 152, 153). As optimal fitness is a moving target, no personality configuration can be beneficial for all purposes, under any circumstances, all the time (16, 48, 154, 184, 185). Instead, selection has pushed organisms toward diversity, so that there is no single “normality” but many (153, 237, 242). On the other hand, much of this variety is not dysfunctional. Some PDs are detrimental for the subject (3, 4), others are not (11, 13), and still others hurt the people all around but benefit the carrier, which is puzzling for a disease (367). As advanced by earlier cognitive theoreticians (368), many PDs seem to be implementing evolved strategies aimed at maximizing biological goals: acquiring mates, outreproducing others, attaining status, garnering resources, or protecting life. They do this with appreciable success, though sometimes at a high cost as well. Accordingly, selective pressures on “pathological” traits are not homogeneously purifying, as would be expected for a disease (15). Instead, some traits are selected for, others against, and still others show tradeoffs (12, 14, 102, 104, 247). Thus, in the eyes of evolution, many PDs are merely unpleasant or socially undesirable conditions (8, 9, 25).

This of course does not imply that PDs are not in need of professional attention. Against the widespread belief that “natural is good” (the naturalistic fallacy), selective pressures do not favor goodness or happiness, but genetic posterity (24, 31). As a result, certain traits are favored by selective forces even if they harm society or the individual, provided that they benefit genes. This results in millions of people living with paralyzing fears, taking absurd risks, or exhausting those whom they love. Against this background, clinicians should be clear that patients do not want to increase fitness, but to relieve pain (369, 370).

## 10. Conclusion

Evolutionary theory is transforming psychology and psychiatry (25); there is a growing awareness that it is essential for the complete understanding of mental conditions (31, 371) and of health and disease more generally (20, 22, 158, 372). The Ukrainian geneticist Theodosius Dobzhansky famously claimed that nothing in biology makes sense except in the light of evolution. PDs certainly do not. Although our knowledge of the selective forces acting on personality is rudimentary (23), we can say for sure that natural selection is the only known mechanism able to produce complex adaptations (18, 373). It follows that personality, like all other body systems, has an evolutionary origin and remains subject to selective forces today, both in humans and in other animals (14, 21, 28, 29, 163, 237). Not only does evolutionary thinking provide the best-substantiated explanatory framework across the life sciences, but it is the conceptual matrix in which different disciplines (genetics, neuroscience, ethology, developmental psychology, and psychopathology) can be integrated (25, 371). Only from this perspective can we truly explain why harmful personalities exist at all, and why they remain over time.

## Author contributions

FG conceived the manuscript and wrote the first draft. FG and FV contributed to the literature review and to the final version of the manuscript. Both authors contributed to the article and approved the submitted version.
